# Comparative Population Structure of Two Deep-Sea Hydrothermal-Vent-Associated Decapods (*Chorocaris* sp. 2 and *Munidopsis lauensis*) from Southwestern Pacific Back-Arc Basins

**DOI:** 10.1371/journal.pone.0101345

**Published:** 2014-07-01

**Authors:** Andrew David Thaler, Sophie Plouviez, William Saleu, Freddie Alei, Alixandra Jacobson, Emily A. Boyle, Thomas F. Schultz, Jens Carlsson, Cindy Lee Van Dover

**Affiliations:** 1 Marine Laboratory, Nicholas School of the Environment, Duke University, Beaufort, North Carolina, United States of America; 2 Nautilus Minerals, Port Moresby, NCD, Papua New Guinea; 3 Environmental Science and Geography Division, School of Natural and Physical Sciences, University of Papua New Guinea, Port Moresby, Papua New Guinea; 4 School of Biology & Environmental Science, University College Dublin, Dublin, Ireland; College of Charleston, United States of America

## Abstract

Studies of genetic connectivity and population structure in deep-sea chemosynthetic ecosystems often focus on endosymbiont-hosting species that are directly dependent on chemical energy extracted from vent effluent for survival. Relatively little attention has been paid to vent-associated species that are not exclusively dependent on chemosynthetic ecosystems. Here we assess connectivity and population structure of two vent-associated invertebrates—the shrimp *Chorocaris* sp. 2 and the squat lobster *Munidopsis lauensis*—that are common at deep-sea hydrothermal vents in the western Pacific. While *Chorocaris* sp. 2 has only been observed at hydrothermal vent sites, *M. lauensis* can be found throughout the deep sea but occurs in higher abundance around the periphery of active vents We sequenced mitochondrial *COI* genes and deployed nuclear microsatellite markers for both species at three sites in Manus Basin and either North Fiji Basin (*Chorocaris* sp. 2) or Lau Basin (*Munidopsis lauensis*). We assessed genetic differentiation across a range of spatial scales, from approximately 2.5 km to more than 3000 km. Population structure for *Chorocaris* sp. 2 was comparable to that of the vent-associated snail *Ifremeria nautilei*, with a single seemingly well-mixed population within Manus Basin that is genetically differentiated from conspecifics in North Fiji Basin. Population structure for *Munidopsis lauensis* was more complex, with two genetically differentiated populations in Manus Basin and a third well-differentiated population in Lau Basin. The unexpectedly high level of genetic differentiation between *M. lauensis* populations in Manus Basin deserves further study since it has implications for conservation and management of diversity in deep-sea hydrothermal vent ecosystems.

## Introduction

In the deep sea, studies of gene flow and population structure have disproportionately focused on numerically dominant taxa from chemosynthetic ecosystems, where it is relatively easy to collect sufficient individuals for population genetic analyses [1]. Within deep-sea chemosynthetic ecosystems, particularly hydrothermal vents, many population genetic studies target holobiont taxa (invertebrate host species with chemoautotrophic symbionts [2]), such as giant tubeworms (*Riftia pachyptila*: [3], [4]) and mussels (*Bathymodiolus thermophilus*: [5]–[7]) on the East Pacific Rise, swarming shrimp (*Rimicaris exoculata* [8], [9]) on the Mid-Atlantic Ridge, or provannid gastropods (*Ifremeria nautilei*:[10], [11] and *Alviniconcha* spp.: [12]) in the western Pacific. Population genetic studies are also reported for holobiont taxa from deep-sea chemosynthetic communities at methane seeps in the Gulf of Mexico and eastern Atlantic [13], [14]. From these studies, we are beginning to understand the extent of genetic connectivity among deep-sea populations associated with chemosynthetic ecosystems and establish natural management units.

Among the first-order questions that arise regarding gene flow and population structure in deep-sea chemosynthetic ecosystems is whether there is greater community and population differentiation among back-arc basins than along mid-ocean ridges, due to the isolated nature of the basins [15], [16]. Vents along mid-ocean ridges tend to be linearly distributed, which may create dispersal corridors [17] where genetic differentiation emerges from geomorphological and hydrographic features rather than distance. In contrast, vents at back-arc basins are non-linearly distributed, forming a complex patchwork of habitat for vent endemic taxa. In the western Pacific, mitochondrial and nuclear microsatellite marker-based population studies in several holobiont taxa suggest genetic homogeneity within back-arc basins (i.e., *Bathymodiolus* spp. [18], [19], *Alviniconcha* spp. [12], [20], *Ifremeria nautilei* [11]). But at larger scales—among back-arc basins—there is evidence for limited connectivity among populations of the provannid gastropods *Alviniconcha* sp. 2 [10] and *Ifremeria nautilei* [10], [11], with populations in Manus Basin isolated from well-connected populations throughout North Fiji and Lau Basins.

Dispersal filters are also observed for vent-associated taxa on the Eastern Pacific Rise (EPR) and often correspond to geomorphological features [3], [7], [21]. These filters tend to be species-dependent, with inconsistent patterns of isolation among taxa. For example, bathymodiolin mussels (*Bathymodiolus thermophilus*) on the East Pacific Rise are divided into northern and southern populations across the Easter Microplate (based on mitochondrial and allozyme markers [7]). *Alvinella pompejana* and *Branchipolynoe symmytilida*, two polychaete species with a similar distributions and one of which (*B. symmytilida*) is commensal in *Bathymodiolus thermophilus*, are undifferentiated across the same boundary (based on a mitochondrial marker [3]). In the northeast Pacific, populations of *Lepetodrilus* limpets diverge across the Blanco Transform Fault, a 450-km long ridge offset that separates the Juan de Fuca and Gorda Ridges (based on mitochondrial and allozyme markers [22]), while unidirectional gene flow was detected in a vent tubeworm, *Ridgeia piscesae*, across the same boundary (based on a mitochondrial maker [23]). Along the Mid-Atlantic Ridge, populations of the mussel *Bathymodiolus puteoserpentis* are connected across approximately 9 degrees of latitude (based on a mitochondrial marker [24]) and hybridize with *Bathymodiolus azoricus* at the Broken Spur vent field (based on mitochondrial and nuclear markers [25]); north of Broken Spur, *B. puteoserpentis* is replaced by *B. azoricus*.

In this study, we explore population structure in two western Pacific back-arc basin vent species—the alvinocarid shrimp *Chorocaris* sp. 2 and the galatheid squat lobster *Munidopsis lauensis*—to determine the extent of population differentiation within and among back-arc basins for vent-associated species. Shrimp in the genus *Chorocaris* are so far only reported at or near hydrothermal vents [26], [27] and *Chorocaris* sp. 2 has, to date, only been observed in Manus and North Fiji Basin (T. Komai, personal communication). Squat lobsters in the genus *Munidopsis* occupy numerous deep-sea habitats, including cold seeps [28], seamounts [29], and wood- and whale-falls [30], and are also generally distributed along the continental slope and bathyal depths [31], [32]. *M. lauensis* is frequently observed in large aggregations near active hydrothermal venting [26], [33], [34], but also occurs in lesser numbers at inactive sulfide edifices on the vent periphery [35] and in still lower abundance as one moves away from the vent field (i.e., >500 m; Thaler, personal observation). Both species are relatively abundant at hydrothermal vents in Manus Basin [34]. Neither species possesses chemoautotrophic endosymbionts, which may make them less dependent on hydrothermal vent fluid than holobiont species, and possibly allows them to use other habitats as stepping-stones for connectivity, though, for both species, larval life history is poorly characterized. We infer that *M. lauensis* is an opportunistic species that is broadly distributed in the deep-sea but is attracted to the rich food source at hydrothermal vents, whereas *Chorocaris* sp. 2 is more patchily distributed and restricted to regions around active hydrothermal venting. We expected that opportunistic vent taxa, like *Munidopsis lauensis*, would show the least population structure, while the vent-associated shrimp, *Chorocaris* sp. 2, would exhibit greater population structure in comparison.

## Materials and Methods

### Sample collection and DNA extraction

Samples were collected with permission of the governments of Papua New Guinea, Fiji, and Tonga. These field studies do not involve any endangered or protected species.

**Figure 1:**
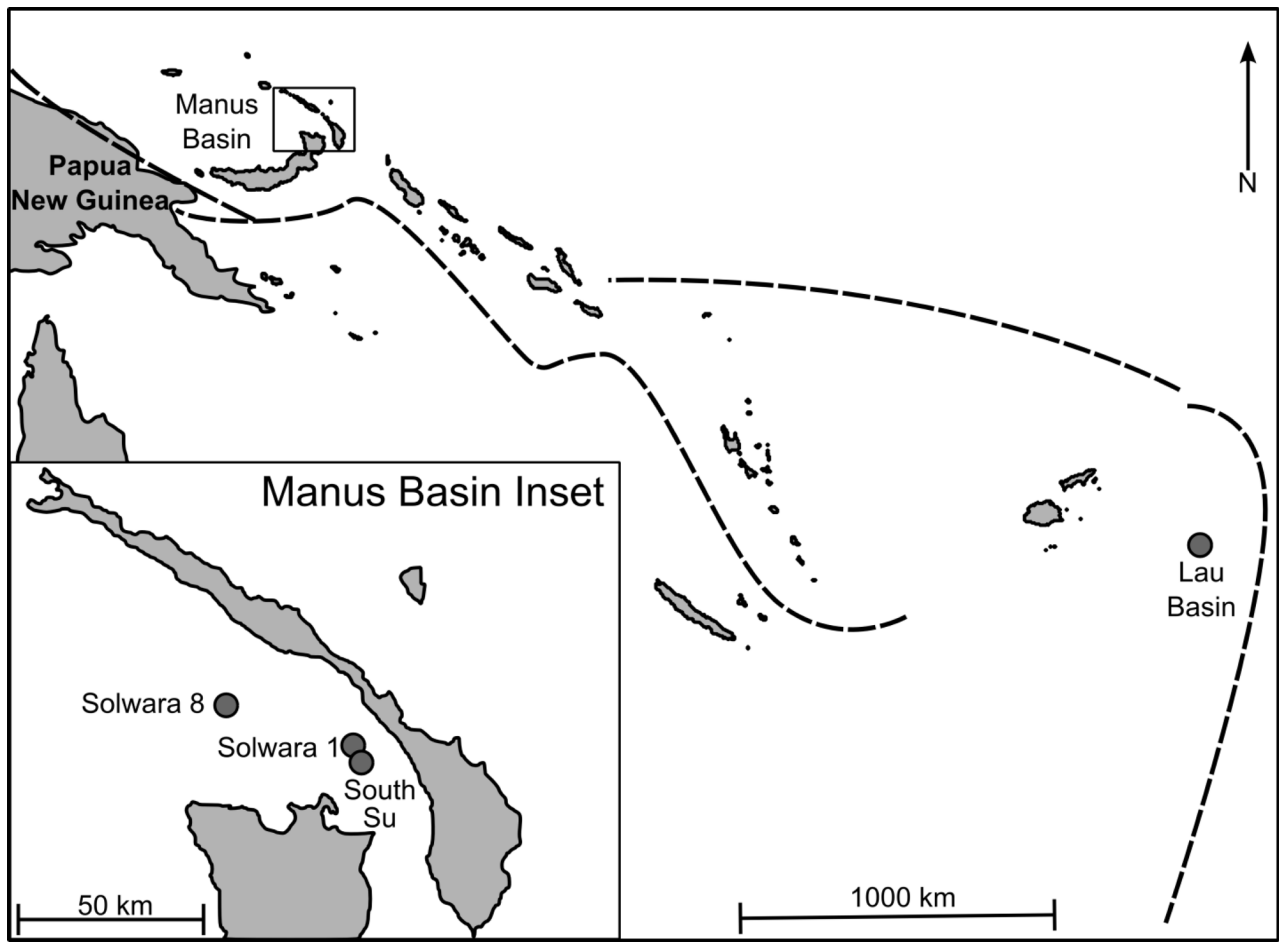
Sampling locations in Manus, North Fiji, and Lau Basins. Dashed lines: subduction zones. Figure originally published in Thaler *et al.* [11].

*Chorocaris* spp. and *Munidopsis lauensis* were collected from three hydrothermal-vent sites in Manus Basin—Solwara 8, Solwara 1, and South Su (Figure 1)—during the *M/V Nor Sky* research campaign in June-July 2008 (Chief Scientist: S. Smith). Samples were collected using an ST200 work class ROV modified for biological sampling. To allow comparisons of population structure at multiple spatial sales, two to four discrete aggregations of *Chorocaris* sp. 2 or *Munidopsis lauensis* were sampled from each site within Manus Basin (Table 1). Additional samples from other southwestern Pacific back-arc basins (Figure 1) were provided by collaborators: *Chorocaris* sp. 2 was collected from Ivory Tower in North Fiji Basin and *M. lauensis* was collected from Hine Hina and Tu'i Malila in Lau Basin in May-June 2005 using the *ROV Jason II* supported by the *R/V Melville* (Chief Scientist: R. Vrijenhoek).

**Table 1:** *Chorocaris* spp. and *Munidopsis lauensis* sampling locations in Manus, North Fiji, and Lau Basin.

	Basin	Site	Mound/Aggregation	Latitude	Longitude	Depth (m)
*Chorocaris* spp.	Manus	Solwara 8	Aggregation 1	3° 43.751′S	151° 40.410′E	1720
			Aggregation 2	3° 43.826′S	151° 40.457′E	1720
			Aggregation 3	3° 43.669′S	151° 40.873′E	1710
		Solwara 1	Aggregation 4	3° 47.453′S	152° 5.485′E	1530
			Aggregation 5	3° 47.367′S	152° 5.781′E	1490
			Aggregation 6	3° 47.372′S	152° 5.619′′E	1480
		South Su	Aggregation 7	3° 48.537′S	152° 6.284′E	1300
			Aggregation 8	3° 48.497′S	152° 6.298′E	1330
			Aggregation 9	3° 48.572′S	152° 6.312′E	1320
			Aggregation 10	3° 48.572′S	152° 6.317′E	1320
	North Fiji	Ivory Tower		16° 59.300′S	173° 54.900′E	1970
*Munidopsis lauensis*	Manus Basin	Solwara 8	Aggregation 1	3° 43.824′S	151° 40.458′E	1710
			Aggregation 2	3° 43.740′S	151° 40.404′E	1720
		Solwara 1	Aggregation 3	3° 47.370'S	152° 05.778′E	1490
			Aggregation 4	3° 47.370′S	152° 05.616′E	1480
		South Su	Aggregation 5	3° 48.564′S	152° 06.144′E	1300
			Aggregation 6	3° 48.492′S	152°0 6.186′E	1350
	Lau Basin	Hine Hina		22° 31.80′S	176° 41.82′W	1900
		Tu'i Malila		20° 59.350′S	176° 34.100′W	1880

Tissues were preserved in 95% ethanol prior to DNA extraction. Genomic DNA was isolated using a standard Chelex-Proteinase-K extraction (10–30 mg digested with 120 µg Proteinase K (Bioline: Taunton, MA) in 600 µl 10% Chelex-100 resin (Bio-Rad: Hercules, CA) overnight at 60°C, heated to 100°C for 15 min, and centrifuged at 10,000 rpm for 5 minutes; [36]) or Wizard SVG tissue extraction kit (Promega Corp: Madison, WI) following manufacturer's protocols. Extracted DNA was stored at 4°C until amplification and archived at −20°C.

### *COI* sequencing and analysis

*Chorocaris* sp 2. mitochondrial *COI* fragments were amplified using the following reaction conditions: 10 to 100 ng of DNA template was combined with 2 µL 10× PCR buffer (200 mM Tris, pH 8.8; 500 mM KCl; 0.1% Triton X-100; 0.2 mg/ml BSA), 2 mM MgCl_2_, 0.6 mM dNTPs, 0.5 µM L*COI*1490 and 0.5 µM H*COI*2198 primers [37], and 1 unit of Taq polymerase in a 20 µL reaction with the following PCR protocol: initial melting temperature of 94°C for 120 seconds; 25 cycles of 94°C for 35 seconds, 48°C for 35 seconds, 72°C for 80 seconds; and a final extension of 72°C for 600 seconds. *Munidopsis lauensis* mitochondrial *COI* fragments were amplified using the following reaction conditions: 10 to 100 ng of DNA template was combined with 2.5 µL PCR buffer, 2 mM MgCl^+^, 0.6 mM dNTPs, 0.05 µM GALA COIR primer (5′-GAA YAG GRT CTC CTC CTC CTA C -3′) and 0.05 µM GALA COIF primer (5′- CAT CAC TWA GWT TRA TYA TTC CAG CAG AA-3′), and 1 unit of Taq polymerase in a 25 µL reaction with the following PCR protocol: initial melting temperature of 94°C for 240 seconds; 35 cycles of 94°C for 60 seconds, 55°C for 120 seconds, 72°C for 210 seconds; and a final extension of 72°C for 600 seconds. Reactions were stored at 4°C until purification.

To remove unincorporated nucleotides, 14 µl of PCR product was incubated with 0.2 µl 10× ExoAP buffer (500 mM Bis-Tris, 10 mM MgCl2, 1 mM ZnSO4), 0.05 µl Antarctic Phosphatase (New England Biolabs: Ipswich, MA), 0.05 µl Exonuclease I (New England Biolabs: Ipswich, MA) at 37°C for 60 min followed by 85°C for 15 min to inactivate the enzymes. Sequencing reactions (both directions) were performed using Big Dye Terminator v3 reactions (Applied Biosystems: Foster City, CA). Dye Terminator removal was performed using AMPure magnetic beads (Agencourt: Morrisville, NC), sequencing products were analyzed on an ABI 3730xl DNA Analyzer (Applied Biosystems International), and sequence chromatograms were edited with CodonCode Aligner (version 3.7.1; CodonCode Corporation: Dedham, MA). Consensus sequences were compared against the NCBI GenBank database to confirm species identity when available [38] and sequence alignments were constructed using the MUSCLE alignment algorithm [39] implemented in CodonCode Aligner. Representative sequences of dominant haplotypes were deposited in GenBank (Accession # KF498731 - KF498847). Full *COI* sequences for each individual are provided as FASTA files (File S1 and S2).

Maximum-parsimony phylograms of aligned mitochondrial sequences were assembled in MEGA version 5 (10,000 replicates; Tamura 3-parameter substitution model determined by Mega 5: Find Best-Fit Substitution Model for both shrimp and squat lobsters; [40]). Potential cryptic species were determined by comparing the degree of divergence between two putative species to the extent of divergence among established species within the same genus [41].

Statistical-parsimony networks were assembled in TCS version 1.21 (default settings; [42]). For *M. lauensis*, 4 *COI* sequences were obtained from NCBI GenBank—EF157850 from the Desmos Caldera in Manus Basin, EF157851 from Mariner vent field in Lau Basin, EF157852 from Hine Hina vent field in Lau Basin, and EF157853 from Brothers Seamount, New Zealand [29]. These sequences were used only for statistical-parsimony network analyses. Number of haplotypes (*H*), haplotype diversity (*Hd*), nucleotide diversity (*π*), and Fu's *F_S_* were calculated using DnaSP version 5.10.01 [43]. DnaSP was also used to construct mismatch distribution curves for expected values under constant population size and population growth/decline models. Arlequin version 3.5.1.2 [44] was used to estimate pairwise *φ_ST_* and permutation tests were used to identify significant departure from genetic homogeneity and Sequential Bonferroni was used to correct for multiple tests [45].

### Microsatellite genotyping and statistical analyses

Six microsatellite markers (*Cho30*, *Cho36*, *Cho63*, *Cho76*, *Cho91*, *Cho99*) were amplified from *Chorocaris* sp. 2 in Manus Basin following methods reported in Zelnio *et al.* [46]. Microsatellite loci were not amplified from *Chorocaris* sp. 2 in North Fiji Basin due to low sample size (n = 9). Nine microsatellite markers (*Mp8, Mp12, Mp14, Mp15, Mp16, Mp21, Mp24, Mp27, Mp29*) were amplified from *Munidopsis lauensis* in Manus and Lau Basin following methods reported in Boyle *et al.* [47]. Full microsatellite genotypes for each individual are provided as GENPOP files (Files S3 and S4).

Divergence from expected Hardy-Weinberg Equilibrium (HWE; GENEPOP; default settings; version 4.0; [48]) and allelic richness (Microsatellite Analyzer; version 4.05; [49]) were assessed. Permutation tests were used to determine significant variation in allelic richness (F-stat; default settings; version 2.9.3.2; [50]). MicroChecker (version 2.2.3; 1000 randomizations; [51]) was used to detect the potential presence of null alleles, stutter, and large allele dropout. To test for the potential influence of selection, loci were screened using LOSITAN (25,000 simulations; IA and SMM; [52], [53]).

Analysis of molecular variance (AMOVA) was used to analyze hierarchal population structure in Arlequin. Pairwise genetic differentiation (*F_ST_*) between aggregations, sites, and basins was analyzed using Microsatellite Analyzer. Alpha levels were adjusted via Sequential Bonferroni to correct for multiple tests [45]. Structure version 2.3.3 (admixture model, sampling locations as prior distributions; [54]) was used to visualize potential population structure. Analyses were conducted with a 1,000,000 step burn-in, 10,000,000 repetitions, and 3 replicates per level from K = 1 to 7. Effective population size was estimated based on microsatellite linkage-disequilibrium using LDNe (default parameters; [55]) and corroborated using ONeSAMP (version 1.2, default parameters [56]). Pairwise relatedness to test for potential kinship effects was estimated with KINGROUP (Version 2_090501; [57]. BOTTLENECK (version 1.2.02.; [58]) was used to test for genetic bottlenecks among site for both *Chorocaris* sp. 2 and *Munidopsis lauensis*. Equilibrium heterozygosity (H_eq_) was estimated under the TPM model allowing for 4% multi-step mutations (1000 iterations).

## Results

### *Chorocaris* sp. 2: Population structure

Samples of shrimp in the genus *Chorocaris* from Manus (191 individuals; Table 2) and North Fiji Basins (9 individuals; Table 2) comprised two putative species based on *COI* genetic divergence (5.4% divergence between species; Figure S1): *Chorocaris* sp. 1 (12 of 41 individuals collected from South Su in Manus Basin), and *Chorocaris* sp. 2 (179 individuals from all three sites in Manus Basin and 9 individuals from North Fiji Basin; Table 2). *Chorocaris* sp. 2, the numerically dominant shrimp species at Manus Basin vents, was further analyzed for population structure using *COI* and microsatellite markers.

**Table 2:** Chorocaris spp. and Munidopsis lauensis.

Species	Location	*N*	*H*	*Hd*	*F_S_*
*Chorocaris* sp. 1	Manus Basin				
	South Su	12	8	0.92	**−3.96**
	Aggregation 7	1	1	n/a	n/a
	Aggregation 9	5	4	0.90	n/a
	Aggregation 10	6	4	0.87	n/a
*Chorocaris* sp. 2	Manus Basin	179	106	0.98	**−162.12**
	Solwara 8	88	60	0.98	**−69.19**
	Aggregation 1	46	37	0.99	**−35.36**
	Aggregation 2	23	20	0.99	**−14.81**
	Aggregation 3	19	15	0.97	**−7.05**
	Solwara 1	62	47	0.98	**−58.13**
	Aggregation 4	14	15	1.00	**−12.00**
	Aggregation 5	5	5	1.00	n/a
	Aggregation 6	43	32	0.98	**−32.95**
	South Su	29	24	0.98	**−17.19**
	Aggregation 7	5	3	0.70	n/a
	Aggregation 8	4	4	1.00	n/a
	Aggregation 9	8	8	1.00	n/a
	Aggregation 10	12	11	0.99	**−4.89**
	Fiji Basin	9	7	0.92	**−1.74**
*Munidopsis lauensis*	Manus Basin	81	4	0.07	n/a
	Solwara 8	43	3	0.09	n/a
	Solwara 1	10	1	0.09	n/a
	South Su	28	2	0.07	n/a
	Lau Basin	30	2	0.07	n/a

Summary statistics for *COI* sequences (*Chorocaris* sp. 2–616 bp; *M. lauensis* –454 bp) from Manus and North Fiji Basins. *N*: number of individuals, *H*: number of haplotypes, *Hd*: haplotype diversity, *F_S_*: Fu's *F_S_*. Significant Fu's *F_S_* indicated in bold. Lau Basin samples are pooled from two sites (Hine Hina and Tu'i Malila) for population comparisons. n/a: index not estimated.

For *Chorocaris* sp. 2, 106 *COI* haplotypes (454 bp) were identified from 179 individuals from Manus Basin. Haplotype diversity among aggregations in Manus Basin was high, ranging from 0.70 to 1.00 (Table 2). Fu's *F_S_* values were all significantly negative (Table 2). Mismatch distribution curves followed unimodal distributions, consistent with a population growth/decline model (Figure S2).

**Figure 2:**
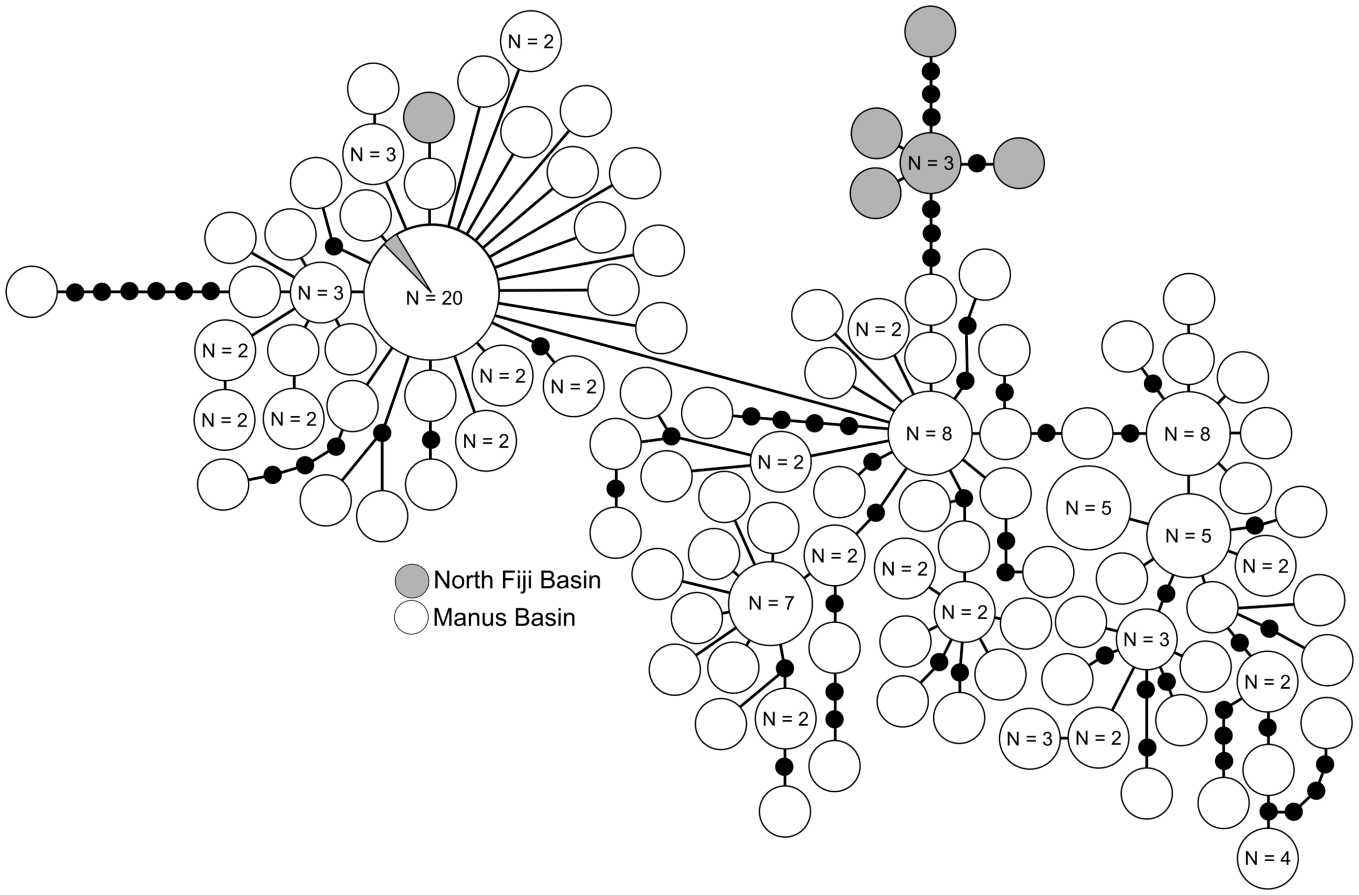
*Chorocaris* sp. 2. Statistical parsimony network for haplotypes from samples collected in Manus and North Fiji Basin. Large circles represent a single individual unless noted on the figure. Small black circles represent inferred haplotypes not observed in this data set. Each node represents 1 pb difference.

The statistical parsimony network for *Chorocaris* sp. 2 has a web-like topology, with many singletons connected through multiple nodes, indicating high genetic variability (Figure 2). A small North Fiji clade branches off the larger Manus clade (Figure 2). One dominant haplotype (n = 20) is shared between Manus and North Fiji Basins and a second North Fiji singleton haplotype is found within the Manus Basin haplotype group (Figure 2). Several additional lineages within Manus Basin are divergent from the main Manus haplotype group by up to 6 mutational steps; this divergence exceeds the *COI*-based genetic divergence found between the main Manus haplotype group and North Fiji Basin haplotype groups (Figure 2).

Six microsatellite loci were amplified from *Chorocaris* sp. 2 within Manus Basin (64 to 92 individuals per site; Table 3). Total alleles per locus ranged from 3 to 11 (mean = 7). In permutation tests, allelic richness (*Rs*) did not vary significantly among patches, mounds, or sites (10,000 permutations, *P*>0.05; Table 3). Neither directional nor balancing selection was detected among microsatellites at any spatial scale (LOSITAN, P>0.05). Three loci deviated significantly from Hardy-Weinberg expectations and showed evidence for heterozygote deficiency at two sites (*Cho63*, *Cho76*, *Cho91*; Table 3). MicroChecker suggested that null alleles were present at all three loci and were responsible for heterozygote deficiencies. As these three markers fell within Hardy-Weinberg expectations when samples from all three sites in Manus Basin are pooled and the presence of null alleles has been shown not to severely bias assignment tests [59], these three markers were used for subsequent analyses.

**Table 3:** *Chorocaris* sp. 2, Manus Basin.

		*Cho30*	*Cho36*	*Cho63*	*Cho76*	*Cho91*	*Cho99*
Solwara 8	*n*	91	91	84	78	89	88
	*a*	3	7	10	9	6	5
	*Rs*	2.66	6.88	9.93	8.90	5.89	4.65
	*as*	185–189	157–189	155–179	232–266	201–213	222–234
	*H_O_*	0.11	0.53	**0.57**	**0.31**	0.21	0.47
	*H_E_*	0.15	0.52	0.65	0.49	0.30	0.46
Solwara 1	*n*	92	81	86	67	89	82
	*a*	4	9	10	10	9	4
	*Rs*	3.76	9.00	9.90	9.90	8.23	4.00
	*as*	183–189	149–189	159–179	248–276	199–215	222–232
	*H_O_*	0.12	0.40	**0.48**	**0.21**	**0.29**	0.41
	*H_E_*	0.11	0.44	0.64	0.45	0.47	0.43
South Su	*n*	90	88	82	64	81	87
	*a*	5	11	8	10	7	6
	*Rs*	5.00	9.59	8.00	10.00	7.00	6.00
	*as*	183–191	145–189	157–173	244–268	201–213	212–234
	*H_O_*	0.21	0.42	**0.37**	**0.20**	**0.16**	0.61
	*H_E_*	0.25	0.47	0.70	0.32	0.39	0.59

Summary statistics for six microsatellite loci Manus Basin. *n*: number of individuals, *a*: number of alleles, *Rs*: allelic richness, *as*: allele size range, *H_E_*: expected heterozygosity, *H_O_*: observed heterozygosity; bold: significant deviation from Hardy-Weinberg expectations after Bonferroni correction for multiple tests.

Analysis of Molecular Variance (AMOVA) for *Chorocaris* sp. 2 and pairwise tests (*F_ST_* and *φ_ST_*) for population differentiation in Manus Basin indicated no significant differentiation at any spatial scale (Table 4). Assignment tests placed all *Chorocaris* sp. 2 from Manus Basin into a single population (Structure, K = 1, data not shown). AMOVA performed on microsatellites across basins indicated that almost 35% of the observed genetic variability was accounted for by differentiation at the basin level, i.e., between Manus and North Fiji Basin (*p*<0.05) and significant pairwise population differentiation was detected between Manus Basin and North Fiji Basin (*φ_ST_* = 0.334 to 0.372; *p*<0.05; Table 4). Effective population size was estimated to be functionally infinite based on microsatellite linkage disequilibrium within Manus Basin samples (based on LDNe and ONeSamp) and KINGROUP indicated no significant pairwise relatedness among individuals within a site. In tests for population bottlenecks, *Chorocaris* sp. 2 departed from mutation-drift equilibrium at Solwara 1 and Solwara 8 (two-tailed *p*<0.05), but not South Su. (two-tailed *p*>0.05) suggesting a recent bottleneck.

**Table 4:** *Chorocaris* sp. 2 and *Munidopsis lauensis* pairwise comparisons of Solwara 8, Solwara 1, South Su, North Fiji, and Lau Basin genetic differentiation.

			Solwara 8	Solwara 1	South Su	Lau Basin
*Chorocaris* sp. 2	Manus Basin	Solwara 8	––	0.005	0.007	––
		Solwara 1	0.006	––	0.013	––
		South Su	−0.009	0.000	––	––
	North Fiji Basin		**0.334**	**0.372**	**0.339**	––
*M. lauensis*	Manus Basin	Solwara 8	––	**0.07**	0.01	**0.12**
		Solwara 1	0.00	––	**0.07**	**0.15**
		South Su	0.00	0.00	––	**0.11**
	Lau Basin		0.00	0.00	0.00	––

Pairwise comparisons of *F_ST_* from microsatellites (above the diagonal), *φ_ST_* from *COI* sequences (*Chorocaris* sp. 2–616 bp; *Munidopsis lauensis* - 454 bp; below the diagonal). Significant genetic differentiation after sequential Bonferroni correction for multiple tests indicated in bold. No microsatellites were deployed on *Chorocaris* sp. 2 collected from North Fiji Basin.

### *Munidopsis lauensis*: Population structure

**Figure 3:**
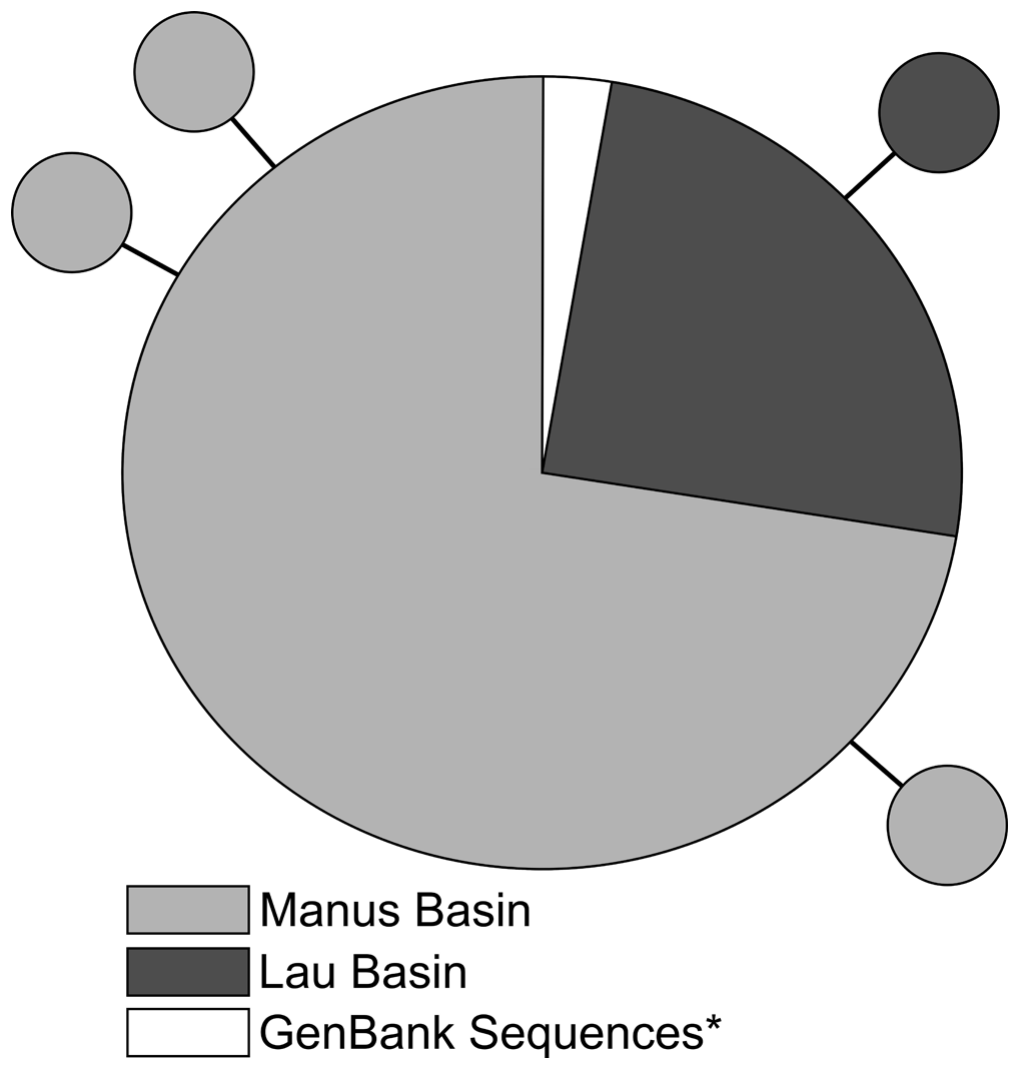
Statistical parsimony network for *Munidopsis lauensis* haplotypes from the western Pacific. Dominant haplotype contains 111 individuals, including four representative sequence recovered from GenBank—Desmos Caldera (Manus Basin; EF157850; [29]), Mariner Vent Field (Lau Basin; EF157851; [29]), Hine Hina (Lau Basin; EF157852; [29]), and Brothers Seamount (New Zealand; EF157853; [29])—indicated with an asterisk. Each node represents 1 bp difference.

A total of 111 *COI* haplotypes (454 bp) were amplified from *Munidopsis lauensis* (81 from Manus Basin, 30 from Lau Basin; Table 2). Three additional individuals from Lau Basin were identified as *Munidopsis antonii*, a closely related species [60]. Haplotype and nucleotide diversity was low in *M. lauensis* (*Hd*<0.09, *π*<0.0002; Table 2): a single *COI* haplotype was present in 107 of the individuals examined. Four singleton *COI* haplotypes were separated by only a single nucleotide mutation from the dominant haplotype (Figure 3). Four sequences obtained from GenBank from two additional sites in Lau Basin, one additional site in Manus Basin, and one from the Brothers Seamount (New Zealand) were identical to the dominant haplotype from our study sites in Manus and Lau Basins (Figure 3).

No *COI*-based genetic differentiation was detected among *Munidopsis lauensis* from Manus or Lau Basin at any spatial scale (*φ_ST_*; Table 4). The low genetic variability of these samples precludes an interpretation of Fu's *F_S_*. AMOVA analyses indicated no hierarchical population structure. Mismatch distribution curves were consistent with a model of stable population size (Figure S2).

Within Manus Basin, all nine microsatellite loci amplified in 17 to 31 individuals of *Munidopsis lauensis* from each site (Table 5). Two loci deviated significantly from Hardy-Weinberg expectations—*Mp24* has an excess of heterozygotes at Solwara 1 and *Mp14* had an excess of homozygotes at South Su (Table 5). No evidence for selection, null alleles, stutter, or large allele dropout was detected. Seven microsatellite loci amplified in both Manus and Lau Basins (*Mp8, Mp14, Mp15, Mp16, Mp24, Mp27, Mp29*), all of which adhered to Hardy-Weinberg expectations (Table 4) with no evidence for selection, null alleles, stutter, or large allele dropout detected. Private alleles were present at all sampling locations, with the highest number of private alleles at Solwara 8 and Solwara 1 (14 each; Table 4) and the lowest number of private alleles at South Su and within Lau Basin (7 each; Table 5)

**Table 5:** *Munidopsis lauensis* microsatellite summary statistics for nine loci Manus and Lau Basin.

Site		*Mp8*	*Mp12*	*Mp14*	*Mp15*	*Mp16*	*Mp21*	*Mp24*	*Mp27*	*Mp29*
Solwara 8	*n*	19	21	26	23	23	17	29	28	28
	*a*	2	8	4	4	1	4	8	5	7
	*Rs*	2.00	7.43	4.00	4.00	1.00	4.00	7.86	5.00	6.61
	*as*	187–228	103–149	155–242	110–190	207–207	252–314	211–278	197–209	203–246
	*H_O_*	0.68	0.52	0.58	0.26	0.00	0.76	0.45	0.54	0.57
	*H_E_*	0.46	0.45	0.49	0.38	0.00	0.62	0.44	0.49	0.65
Solwara 1	*n*	28	24	30	25	29	29	29	31	30
	*a*	2	5	6	4	6	9	6	5	8
	*Rs*	2.00	4.58	5.85	4.00	5.70	7.37	5.90	4.81	7.66
	*as*	187–228	103–245	155–244	110–190	207–447	152–314	127–229	197–212	206–246
	*H_O_*	0.32	0.46	0.57	0.72	0.38	0.90	**0.97**	0.32	0.63
	*H_E_*	0.27	0.39	0.74	0.71	0.34	0.72	**0.66**	0.41	0.82
South Su	*n*	19	19	30	29	28	25	28	29	23
	*a*	2	2	6	5	6	7	4	4	5
	*Rs*	2.00	2.00	5.73	4.79	5.29	6.03	4.00	3.97	5.00
	*as*	187–228	103–143	155–244	110–190	160–446	238–326	129–229	197–206	229–246
	*H_O_*	0.58	0.42	**0.33**	0.41	0.32	0.84	0.25	0.48	0.65
	*H_E_*	0.42	0.34	**0.57**	0.41	0.29	0.74	0.29	0.52	0.60
Lau Basin	*n*	29	––	35	31	34	––	36	32	37
	*a*	2	––	4	2	10	––	3	4	3
	*Rs*	2.00	––	3.74	1.74	8.16	––	2.78	4.00	6.62
	*as*	187–228	––	155–242	103–110	207–446	––	127–219	197–209	226–244
	*H_O_*	0.17	––	0.40	0.03	0.44	––	0.89	0.34	0.16
	*H_E_*	0.16	––	0.47	0.03	0.43	––	0.51	0.38	0.24

*n*: number of individuals, *a*: number of alleles, *Rs*: allelic richness, *as*: allele size range, *H_E_*: expected heterozygosity, *H_O_*: observed heterozygosity; bold: significant deviation from Hardy-Weinberg expectations after sequential Bonferroni correction for multiple tests.

**Figure 4:**
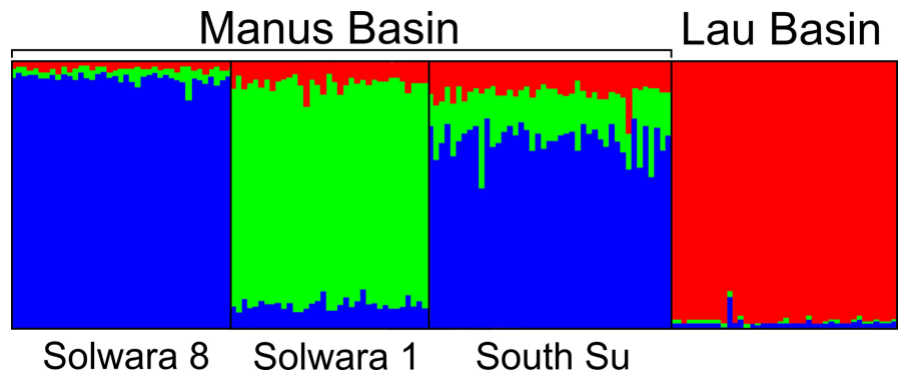
*Munidopsis lauensis* structure output for seven microsatellite loci shared across Manus and Lau Basin. Each color represents a different putative population inferred from the distribution of allele frequencies. K = 3 was determined to be the most likely model based on 5 replicates each of model runs from k = 1 to k = 7, with a 1,000,000 step burn-in period followed by 10,000,000 steps. Sampling locations were used as priors for putative population assignments.

Pairwise tests for genetic differentiation based on seven microsatellite markers that amplified in samples from all sites in Manus as well as Lau Basin revealed significant genetic differentiation between Solwara 1 and the other two sites (South Su and Solwara 8) in Manus Basin (*F_ST_* = 0.07; *p*<0.05; Table 4) and significant genetic differentiation between populations of *Munidopsis lauensis* in Lau Basin and those in Manus Basin (*F_ST_* ≥ 0.11; *p*<0.05; Table 4). AMOVA analyses indicated that between-basin effects accounted for nearly 90% of the hierarchal population structure; within-Manus effects accounted for ∼10% of the structure. Assignment tests (Structure) suggested that the most likely number of populations is 3 (K = 3, average ln P(D) = −1489.6), with one population in Lau Basin, a second population at Solwara 1 in Manus Basin, and a third population shared at Solwara 8 and South Su in Manus Basin (Figure 4). Effective population sizes for both populations in Manus Basin as well as the Lau Basin population were estimated to be functionally infinite based on microsatellite linkage disequilibrium (based on both LDNe and ONeSamp). KINGROUP indicated no significant pairwise relatedness among individuals within sites in Manus Basin or within Lau Basin. No significant bottleneck effects were detected for *M. lauensis* as all ‘sites’ were found to be in mutation-drift equilibrium.

## Discussion

### Overview

Genetic differentiation of species endemic to discrete habitats tends to be positively correlated with the degree of patchiness of those habitats, especially in species with limited dispersal potential [61], though lack of genetic differentiation over large spatial scales has been observed for vent taxa and inferred to be a consequence of the ephemeral nature of vent patches [16]. Genetic differentiation may also be reduced in species associated with patchy habitats that can also exploit alternative habitats, albeit in lower densities (*e.g.*, coral-reef fish display increased gene flow when populations are connected via intermediate, non-coral-reef ‘stepping stones’, [62]). While we expected that the opportunistic *Munidopsis lauensis* would have less population structure when compared with *Chorocaris* sp. 2, we discovered that *M. lauensis* exhibited strong signals of genetic differentiation at relatively small spatial scales, whereas the population structure of *Chorocaris* sp. 2 was similar to other vent-associated species from the western Pacific (i.e., *Ifremeria nautilei* [11]).

### Cryptic Species

Cryptic or miss-identified species were discovered in samples of shrimp and squat lobsters from western Pacific back-arc basins. *Chorocaris* sp. 1, a shrimp closely related to *Chorocaris* sp. 2 [46], was identified from *COI* sequences of shrimp from South Su (Manus Basin). *Chorocaris* sp. 1 is not known from other sites in the western Pacific (T. Komai, personal communication). Three individuals of *Munidopsis antonii*, a squat lobster closely related to *M. lauensis* [31] were found in Lau Basin samples. *M. antonii* is broadly distributed [31], but this is the first report of the species in Lau Basin. Cryptic species have the potential to confound population genetics studies by introducing divergent genetic diversity into analyses [41], thus identifying and excluding cryptic or misidentified species from population samples is an important first step in any population study where morphological identifications are challenging.

### *Chorocaris* sp. 2: Population structure

The homogeneous distribution of *Chorocaris* sp. 2 haplotypes and microsatellite markers within Manus Basin is consistent with high gene flow among the study sites. Although larvae are likely the primary dispersal vector, the mobility of juvenile and adult shrimp may also allow individuals to travel among neighboring vent sites, reducing the potential for local population structure to emerge.

There is a high frequency of rare, private haplotypes (78.3% are singletons) from Manus and North Fiji Basin samples of *Chorocaris* sp. 2. The excess of rare *COI* haplotypes (significantly negative Fu's *F_S_*) and departure from mutation-drift equilibrium for microsatellite markers at Solwara 1 and Solwara 8 suggest that these two populations encountered a recent bottleneck followed by a population expansion. South Su also departed from mutation-drift equilibrium based on *COI* (*F_S_* values are significantly negative but higher than for the other populations) but not for microsatellite markers. This could indicate that the bottleneck encountered by the South Su population was not as strong as bottlenecks affecting other populations, or that the recovery from the bottleneck was faster in South Su. Bottlenecks followed by population expansions have been found in other species [25], including in the *Rimicaris exoculata* shrimp on the Mid-Atlantic Ridge [8], [9] and in other hydrothermal vent-associated species from Manus Basin [11], [63]. Although null alleles were found at three loci, the presence of null alleles results in inflated estimates of populations differentiation [59]. Given that no genetic differentiation was detected among *Chorocaris* sp. 2 sampled from multiple sites in Manus Basin, the null alleles did not influence the overall outcome.

Despite strong signals of genetic differentiation in *COI* sequences between populations of *Chorocaris* sp. 2 from Manus and North Fiji Basins, the presence of two shared *COI* haplotypes between basins indicates that some migration must occur or have occurred in the recent past or that the populations are experiencing incomplete lineage sorting. The absence of *COI* haplotypes descended from the North Fiji clade in Manus Basin suggests that migration, if it occurs, may be directional, from Manus into North Fiji Basin, though this could be the result of sample bias, given the small number of individuals (n = 9) sampled from North Fiji. A Manus to North Fiji route is consistent with the regional circulation patterns and recent models of larval transport in the southwest Pacific [64].

In contrast to *Chorocaris* sp. 2, *COI* and microsatellite-based population studies of the related *Rimicaris exoculata* from hydrothermal vents along the Mid-Atlantic Ridge found no population structure across more than 5,000 kilometers [8], [9]. Haplotype diversity in *R. exoculata* populations (*Hd* = 0.69 to 1.00) is similar to populations of *Chorocaris* sp. 2 (*Hd* = 0.70 to 1.00). Teixeira *et al.* [8] suggest that the *R. exoculata* population is the product of a recent founder event followed by demographic expansion along the Mid-Atlantic Ridge, while population structure of *Chorocaris* sp. 2 appears to arise from a barrier to gene flow from basin to basin in the southwestern Pacific.

### *Munidopsis lauensis*: Population structure

*COI* haplotype diversity in *Munidopsis lauensis* is low (*Hd* = 0.07–0.09) and is an order of magnitude lower than that observed in other vent-associated species in Manus Basin ([11], Van Dover laboratory, unpublished data). The 96.4% dominance of a single *COI* haplotype in *M. lauensis* contrasts with that of other species from western Pacific deep-sea hydrothermal vents [16] but is consistent with that of *M. polymorpha* from an anchialine pool in the Canary Islands [65]. A low *COI* mutation rate is characteristic of related squat lobsters [60], [66]. Alternatively, a recent selective sweep could have reduced the number of haplotypes in the populations [67] though it could also be indicative of a recent population expansion [68]. This interpretation is not supported by the mismatch distribution curves, which indicate a stable population size. For a selective sweep to reduce haplotype diversity in *M. lauensis* populations in both Manus and Lau Basins to a single dominant haplotype, these populations must be or once have been well-connected, otherwise other common haplotypes would have propagated in the isolated populations [69]. The processes that produce low observed haplotype diversity are, as yet, undetermined. However, all interpretation of *M. lauensis* mitochondrial population structure are necessarily constrained by the limited variability of *COI* haplotypes.

Past microsatellite-based studies of galatheid squat lobster population structure have been confounded by the presence of mobile, cryptic, microsatellite-flanking transposable elements in squat lobster genomes [70]. Transposable elements were observed in three squat lobster species (*Munida rugosa*, *Munida sarsi*, and *Galatheae strigosa*), causing inconsistencies and failures in microsatellite amplification and amplification of multiple fragments [70]. The symptoms of these elements were not reported in microsatellites developed for *Munidopsis polymorpha* [71], nor were they observed in the amplification and analysis of *Munidopsis lauensis* for this study.

Microsatellite-based estimates of genetic differentiation in *Munidopsis lauensis* yielded evidence for fine-scale population structure among sites in Manus Basin, but not within sites. *M. lauensis* from Solwara 1 are genetically differentiated from those of South Su (only ∼2.5 kilometers apart) and Solwara 8 (∼40 km apart), but *M. lauensis* from South Su and Solwara 8 (∼40 km apart) are genetically undifferentiated. While family effects—the appearance of genetic differentiation due to a region being colonized by closely related propagules—could explain the observed genetic differentiation between Solwara 1 and the other Manus Basin sites, no indication of significant relatedness among individuals from Solwara 1 was detected. Isolation of *M. lauensis* at Solwara 1 could be the result of physical or hydrographic barriers to gene flow that limit colonization or migration from other sites in Manus Basin. Solwara 1 lies along the northwest flank of a large, submerged, and active volcano (North Su), which physically separates Solwara 1 from South Su [72]. In addition, the St. George's Undercurrent runs roughly northwest through Manus Basin, passing first over South Su, then over Solwara 1 and Solwara 8 [73]. If *M. lauensis* possesses larva that remain near the sea floor, the path of the St. George's Undercurrent could prevent *M. lauensis* larvae from effectively dispersing up-current, from Solwara 1 southwestward to South Su.

*Munidopsis lauensis* is genetically differentiated between Manus and Lau Basins based on microsatellite data, but the less variable *COI* sequences do not detect this differentiation. These divergent results from two types of genetic markers highlight one of the challenges in conservation genetics. Because multiple phenomena likely shape the genetic diversity and distribution of *M. lauensis* throughout the western Pacific, no single gene, or suite of similar genetic loci (e.g., mitochondrial or microsatellite), can provide a complete picture of population structure [74]. Our interpretation is that different genes represent different processes in *M. lauensis*. On an evolutionary time-scale, *COI* data suggest that homogenizing processes (gene flow, selective sweeps, or lack of time for mutations to accumulate after a founder event) have reduced the genetic diversity of *M. lauensis*. The lack of a recent population bottleneck detected in the microsatellites supports a selective sweep on *COI*. On an ecologic time-scale, microsatellite data suggest locally differentiated populations and restricted gene flow over relatively short distances (2.5 km); the extent to which locally differentiated populations persist over multiple generations of *M. lauensis* is unclear and requires an assessment of temporal variability among these differentiated populations. A similar phenomenon of divergent estimates of population structure based on differing marker types was observed among species in the tubeworm genus *Escarpia* that inhabit cold seeps in the Gulf of Mexico, Gulf of California, and West Africa; no significant population differentiation was observed based on mitochondrial markers, but microsatellites revealed some significant differentiation among regions [75].

When a suite of microsatellite loci is developed for one particular population, it may not work as well in additional distantly related populations, resulting in null alleles [76]. For *M. lauensis*, this kind of ascertainment bias is apparent in two microsatellite loci, *Mp12* and *Mp21* (as these markers were developed on *M. lauensis* from Manus Basin), which failed to amplify in individuals from Lau Basin. It is possible that these loci are not present in Lau Basin samples. The frequency of alleles for the other seven markers fell within Hardy-Weinberg expectations, with no evidence of null alleles, and suggests that they are not influenced by ascertainment bias. The failure of two microsatellites to amplify, suggests that the observed genetic divergence between *M. lauensis* from Manus and Lau Basin may be even more pronounced.

### Isolation of Manus Basin Vent Fauna

There is strong genetic evidence that *Chorocaris* sp. 2 and *Munidopsis lauensis* in Manus Basin are isolated from other back-arc basin vent systems. Three provannid snail species (*Ifremeria nautilei*, *Alviniconcha* sp.1, and *Alviniconcha* sp. 2) also exhibit strong population isolation between Manus Basin and other regional back-arc basins (North Fiji, Lau Basins, Marianna Trough [11], [12], [20], [77]). Barriers to dispersal have been documented for other vent taxa in the eastern Pacific, where the barriers are often associated with geomorphological features (*e.g.* Easter Microplate: [3], [7]; Blanco Transform Fault: [22], [23]; or hydrodynamic gyres: [3]). Hydrothermal vents in the eastern Pacific are distributed in a roughly linear pattern [17], [78], with population differentiation often occurring along a north/south gradient [17], [21], [23], [25], [78], [79]. The presence of the New Guinea archipelago may create a physical barrier to migration out of Manus Basin [11], while the north-westward path of the St. George's undercurrent may provide a limited pathway for propagules to disperse from Manus eastwards towards North Fiji and Lau Basins [73]. Larval transport models based on an assumption of long-lived, lecithotrophic, deep-sea larvae indicate that, even after 500 days of dispersal, few larvae would be transported into Manus Basin from surrounding regions [64].

### Implications for conservation and management

Characterization of patterns of genetic diversity and connectivity within and among populations is a valuable tool for managing and mitigating the effects of anthropogenic disturbance at deep-sea hydrothermal vents [80]. In Manus Basin, Solwara 1 has been identified as a site for mineral extraction, while South Su has been set aside as a refuge [72]. Key vent-associated species shared between South Su and Solwara 1 (*Chorocaris* sp. 2 and *Ifremeria nautilei*) have a high degree of connectivity, providing evidence that South Su may serve as an effective reservoir of genetic diversity for some species. For *Munidopsis lauensis*, however, Solwara 1 and South Su populations are genetically distinct, and, in this case, South Su would not act as an effective reservoir of genetic diversity. Population differentiation between *Munidopsis lauensis* from Solwara 1 and other sites is perplexing. If the squat lobsters are genetically isolated through some as yet-to-be-determined mechanism, it is not clear that this genetic lineage could be sustained in the face of severe population reduction. Genetic differentiation at such a local scale, however, is a surprising outcome for this species and requires further investigation.

There is limited connectivity between the Manus Basin populations *Chorocaris* sp. 2 and *Munidopsis lauensis* and conspecific populations in other western Pacific back-arc basins. This suggests that if maintenance of genetic diversity is an environmental management objective, then management tools must be applied on a regional basis (e.g., within Manus Basin) for these species. Ongoing monitoring of genetic diversity of key taxa before and after mineral extraction would allow managers to assess the impact of the activity on connectivity and population structure and inform best practices.

## Supporting Information

Figure S1 **Maximum likelihood tree for a subset of** ***Chorocaris*** **spp. sampled from Manus and North Fiji Basin.** Sequences are 600-base pairs in length. Substitution model is Tamura 3-parameter determined by Find Best Model application in Mega 5. Representatives of *Chorocaris* sp. 1 and sp. 2 were chosen at random. *Chorocaris* sp. 1 and *Chorocaris* sp. 2 indicated by horizontal bars. *Chorocaris vandoverae* (Mariana Trough; accession # AF125417; [81]) and *Rimicaris exoculata* (Mid-Atlantic Ridge; accession # FN393000) presented for comparison. Bootstrap values greater than 0.50 reported on branches. Scale bar is number of substitutions per base pair.
(TIF)

Figure S2 **Observed and expected mismatch curves of pairwise mitochondrial** ***COI*** **nucleotide differences for** ***Chorocaris*** **sp. 2 sampled from Manus and North Fiji Basin and** ***Munidopsis lauensis*** **sampled from Manus and Lau Basin.** Each graph represents a comparison between simulated curves for pairwise nucleotide differences and observed pairwise differences for (A) *Chorocaris* sp. 2 under a model of constant population size, (B) *Chorocaris* sp. 2 under a model of population growth and decline, (C) *Munidopsis lauensis* under a model of constant population size, and (D) *M. lauensis* under a model of population growth and decline.
(TIF)

File S1 **FASTA format file for all** ***Chorocaris*** **spp.** ***COI*** **sequences.**
(NEX)

File S2 **FASTA format file for all** ***Munidopsis lauensis COI*** **sequences.**
(NEX)

File S3 **GENPOP format file of all** ***Chorocaris*** **spp. microsatellite markers.**
(XLS)

File S4 **GENPOP format file of all** ***Munidopsis lauensis*** **microsatellite markers.**
(XLSX)
