# Supplementary figures and images for: Comparative Population Structure of Two Deep-Sea Hydrothermal-Vent-Associated Decapods (Chorocaris sp. 2 and Munidopsis lauensis) from Southwestern Pacific Back-Arc Basins
Source: PLoS One. 2014 Jul 1;9(7):e101345. doi: 10.1371/journal.pone.0101345 (PMC4077841; doi:10.1371/journal.pone.0101345)

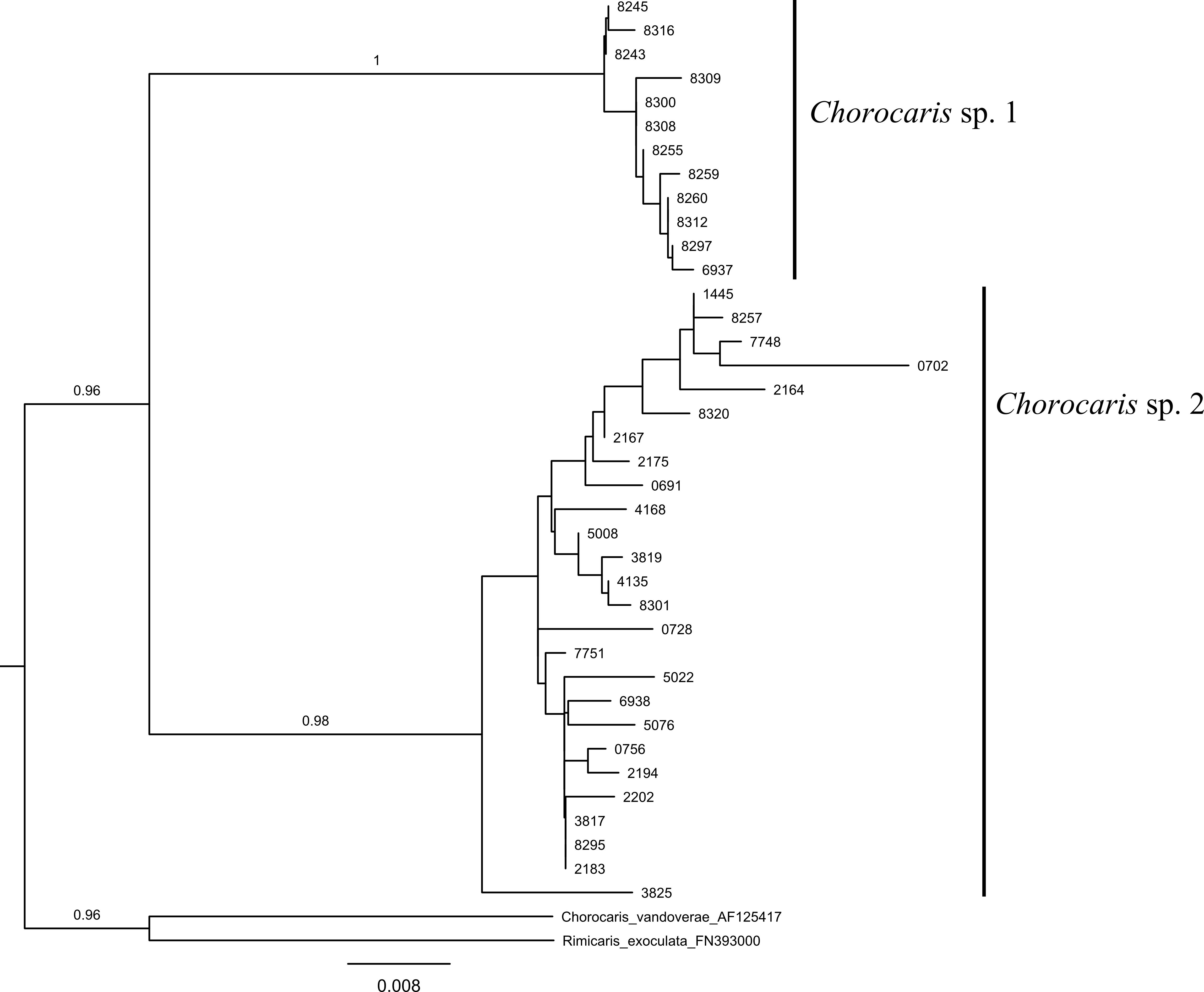

Supplement: Figure S1 — Maximum likelihood tree for a subset of Chorocaris spp. sampled from Manus and North Fiji Basin. Sequences are 600-base pairs in length. Substitution model is Tamura 3-parameter determined by Find Best Model application in Mega 5. Representatives of Chorocaris sp. 1 and sp. 2 were chosen at random. Chorocaris sp. 1 and Chorocaris sp. 2 indicated by horizontal bars. Chorocaris vandoverae (Mariana Trough; accession # AF125417; [81]) and Rimicaris exoculata (Mid-Atlantic Ridge; accession # FN393000) presented for comparison. Bootstrap values greater than 0.50 reported on branches. Scale bar is number of substitutions per base pair. (TIF) [file pone.0101345.s001.tif]

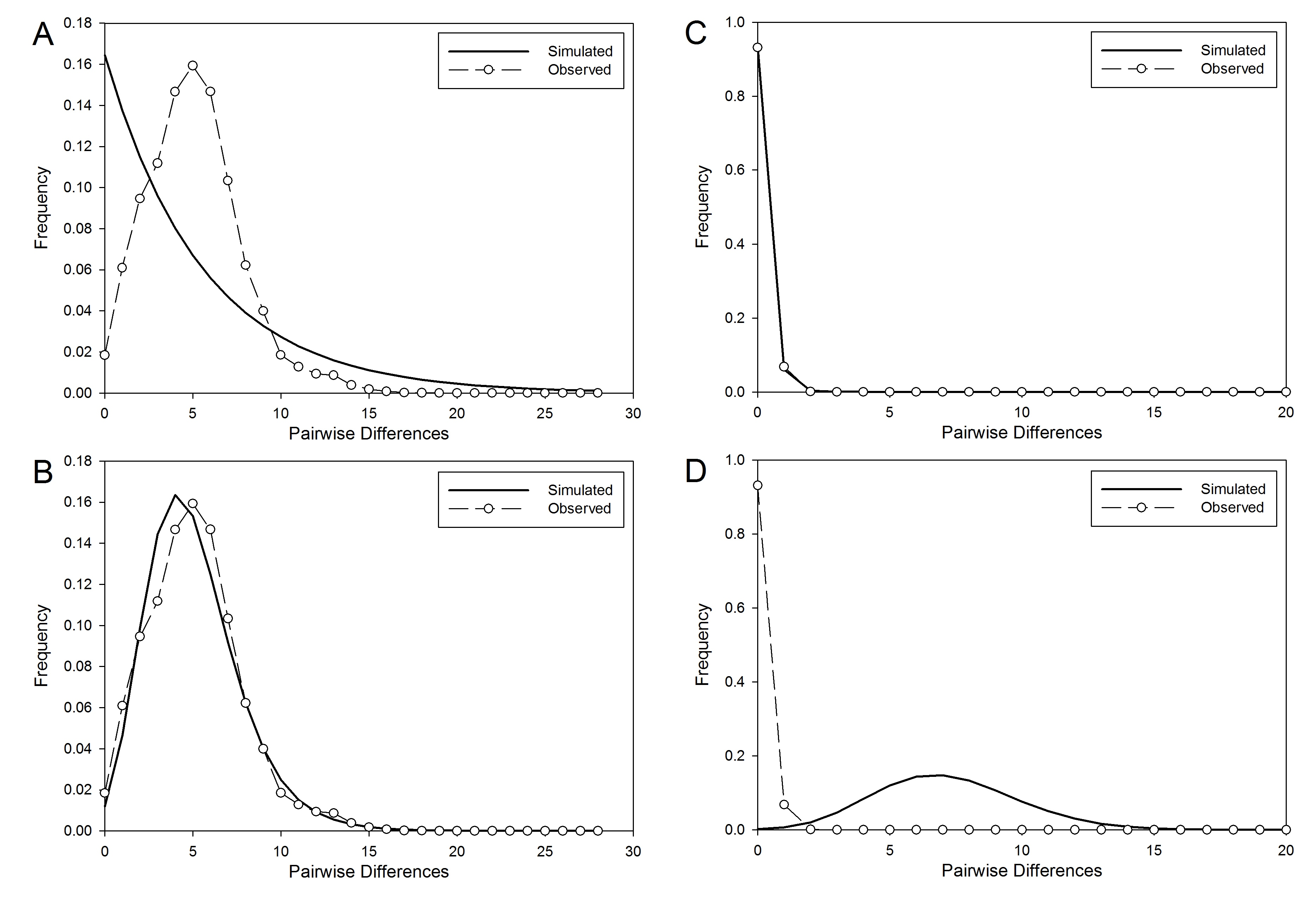

Supplement: Figure S2 — Observed and expected mismatch curves of pairwise mitochondrial COI nucleotide differences for Chorocaris sp. 2 sampled from Manus and North Fiji Basin and Munidopsis lauensis sampled from Manus and Lau Basin. Each graph represents a comparison between simulated curves for pairwise nucleotide differences and observed pairwise differences for (A) Chorocaris sp. 2 under a model of constant population size, (B) Chorocaris sp. 2 under a model of population growth and decline, (C) Munidopsis lauensis under a model of constant population size, and (D) M. lauensis under a model of population growth and decline. (TIF) [file pone.0101345.s002.tif]
